# Description and characterization of the artisanal elasmobranch fishery on Guatemala’s Caribbean coast

**DOI:** 10.1371/journal.pone.0227797

**Published:** 2020-01-13

**Authors:** Ana Hacohen-Domené, Francisco Polanco-Vásquez, Colombo Estupiñan-Montaño, Rachel T. Graham

**Affiliations:** 1 Fundación Mundo Azul, Blvd. Rafael Landívar,Guatemala City, Guatemala; 2 Fundación Alium Pacific, Cali, Colombia; 3 MarAlliance, San Pedro, Belize; Australian Bureau of Agricultural and Resource Economics and Sciences, AUSTRALIA

## Abstract

Small-scale shark and ray fisheries are conducted throughout Central America’s Caribbean coast. Yet, there is limited information regarding catch composition and diversity of these fisheries, especially in Guatemala. Surveys of catch landings were conducted in two of Guatemala’s primary Caribbean coastal shark and ray fishing communities, El Quetzalito and Livingston, between January 2015 and July 2017. Biological data from 688 landed chondrichthyans were collected, with 31 species (24 sharks, six rays and one chimaera) identified. The four most frequently captured species included *Carcharhinus falciformis* (30.2%), *Sphyrna lewini* (12.7%), *Hypanus guttatus* (12%) and *Rhizoprionodon* spp. (6.7%). Landed sharks contained most size classes with a high proportion of juveniles of species with low productivity. The large-bodied species *C*. *falciformis* and *S*. *lewini* were often recorded at sizes below known maturity; 96.6% and 85.1%, of the captured individuals were immature, respectively. This study can serve as a baseline to determine future trends in the elasmobranch fisheries conducted by Guatemala’s Caribbean coastal communities and support assessments on the persistence of the fisheries.

## Introduction

Threats to the sustainability of elasmobranch populations are of increasing concern to scientists and fisheries managers who catalogue linkages between fisheries exploitation and declining shark and ray populations in various regions of the world [1]. Shark fisheries are increasing as demand rises for shark products and the meat increasingly represents an important source of food security in many countries while targeted finfish species populations decline (e.g. [2,3]). The Food and Agriculture Organization of the United Nations (FAO) reports that chondrichthyan landings increased steadily until 2003, but have decreased by 20% since [1,4]. However, total catch may be 3–4 times higher than reported, as many shark fisheries are illegal, unregulated and/or unreported [5]. A lack of species-specific landings information further confounds the collection of catch statistics due to a lack of recording, misidentification of species or discards at sea [4].

Increasing awareness and concern of the current status of sharks and rays have driven the protection of threatened species such as large-bodied hammerheads (*Sphyrna* spp.) through recently enacted national and international measures [6,7,8]. Yet, landings data for chondrichthyans remain limited or unavailable in many regions, including the Caribbean, undermining population assessments and science-based management measures. These data gaps further limit countries that attempt to meet their international convention obligations such as the Convention for the International Trade in Endangered Species of Fauna and Flora (CITES). Studies of Caribbean-ranging shark species and fisheries are increasing [9], but remain highly site-specific and research has focused on more predictable or commonly encountered species, such as the Caribbean reef shark *Carcharhinus perezi* [10,11] and the whale shark *Rhincodon typus* [12,13].

FAO reports that shark landings in the Western Central Atlantic are dominated by the genus *Carcharhinus* and that by the year 2006, elasmobranch captures were calculated to be about 6,344 metric tons [9]. The Mesoamerican Reef (MAR) region extends more than 1,000 km from the north-eastern tip of Mexico’s Yucatan Peninsula southward through Quintana Roo, the territorial waters of Belize, Guatemala and northern Honduras. In Quintana Roo, shark captures are dominated by the Atlantic sharpnose shark *Rhizoprionodon terraenovae*, the bull shark *Carcharhinus leucas* and the nurse shark *Ginglymostoma cirratum* [14,15]. In Belize, Graham [16] and Zeller et al. [17] report that the Caribbean sharpnose shark *Rhizoprionodon porosus*, the great hammerhead shark *Sphyrna mokarran*, the scalloped hammerhead *Sphyrna lewini*, *G*. *cirratum and C*. *leucas* represent an estimated 90% of the catch. In Honduras, Morales et al. [18] report that the bonnethead shark *Sphyrna tiburo*, *Sphyrna* spp., *C*. *leucas*, *Carcharhinus signatus* and *R*. *porosus* were the main shark species landed. In 2011, Honduras declared its waters a permanent shark sanctuary, banning all shark fishing (Decreto No. 107–2011, Art. 1), which curtailed elasmobranch fisheries-dependent data collection. However, it should be noted that in 2016, Honduras legalized the fishing of sharks that are incidentally captured (Decreto No. 26–2016).

On Guatemala’s Atlantic coast, shark and ray fisheries represent one of several economic activities that generate jobs and provide food to coastal communities. In these communities, shark and ray fisheries are small-scale artisanal fisheries that use small boats of 6–7 m length fitted with outboard motors. Gears of choice include longlines and gillnets. Bigelow and Schroeder [19], the first report on elasmobranchs in Guatemala, listed two female *C*. *leucas* (692 mm and 920 mm total length) collected on a survey done by the U.S. Fish and Wildlife Service in the Caribbean. In addition to Bigelow and Schroeder [19], there are a few first records of elasmobranch species range extensions in Guatemala’s Caribbean waters [20–22].

Baseline and species-specific data are largely unavailable for artisanal elasmobranch fisheries, but are essential for the monitoring of exploited populations and the development of effective management plans [23]. Considering the migratory nature of some species landed in Guatemala, connectivity with other sites along the MAR is plausible [24]. So, by conducting this study we intend to support the development of regional analyses and conservation and management measures in the region.

Our aims were 1) to provide a baseline on chondrichthyan fisheries catch occurring along Guatemala’s Caribbean coast, based on a survey of landings composition from 20 fishing vessels at the two major ports on the Caribbean coast of Guatemala; 2) to provide biological information for the most frequently captured species; and 3) to generate a current taxonomic list of chondrichthyans along Guatemala’s Caribbean coast.

## Materials and method

### Study site description

This project was carried out under research permit number DRNOR0012016, issued by the Consejo Nacional de Áreas Protegidas (CONAP), Guatemala. Guatemala is located in Central America, bordering the Caribbean Sea, with approximately 140 km of coastline (between Belize and Honduras) and bordering the Northern Pacific Ocean (between Mexico and El Salvador) with approx. 255 km of coastline.

**Fig 1:**
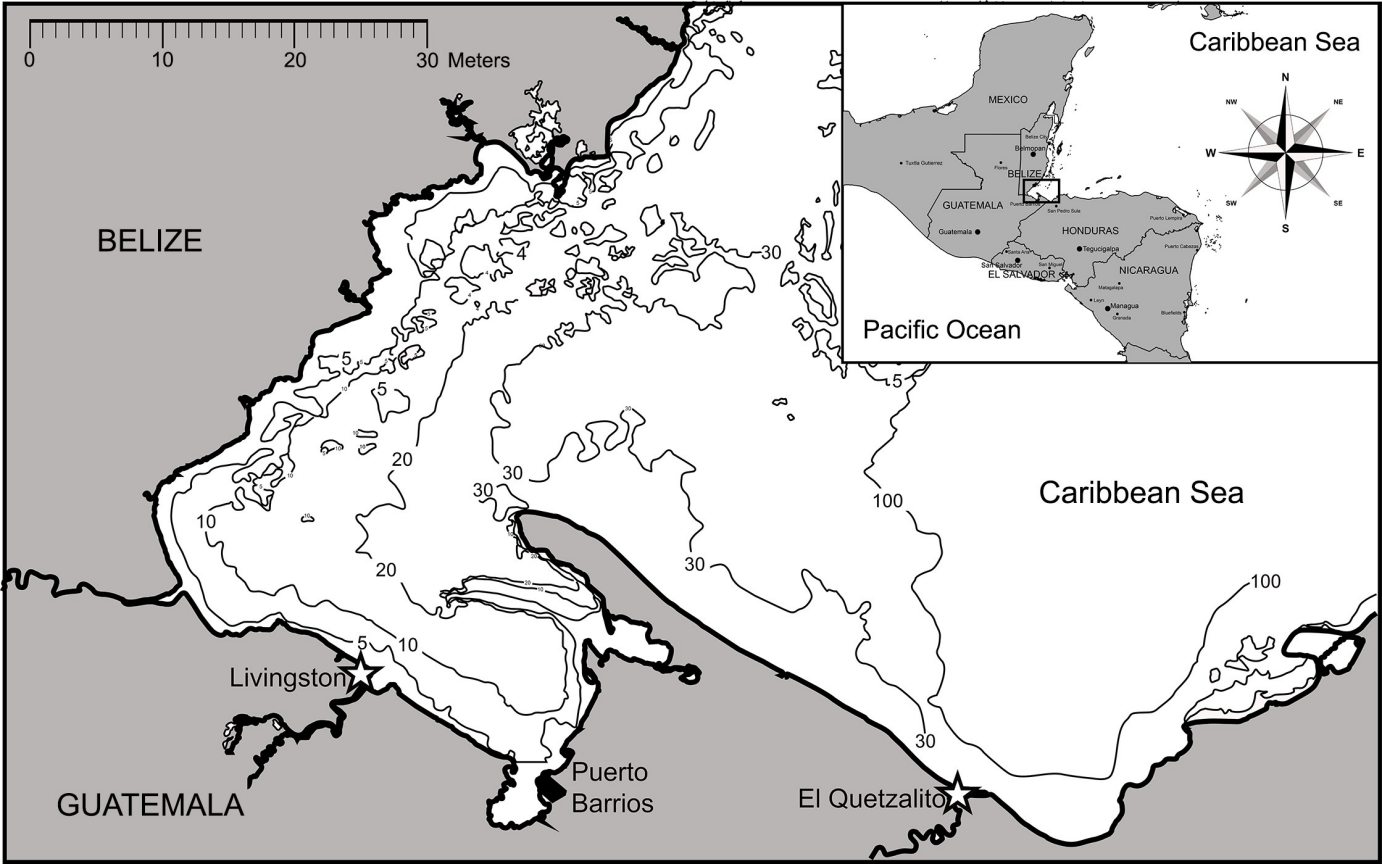
Study area showing location (☆) where landings monitoring was conducted.

For this study, we conducted surveys from January 2015 to July 2017, at landing sites in two fishing communities along Guatemala’s Caribbean Sea: El Quetzalito (15°43’34.72” N, 88°17’25.38” W) and Livingston (15°49’27.17” N, 88°45’1.71” W) (Fig 1). Fishers from these communities land the majority of elasmobranchs captured along the coast. El Quetzalito is nestled inside the Wildlife Refuge of Punta Manabique (Refugio de Vida Silvestre Punta de Manabique), in the area known as Barra Motagua. Principal economic activities in the area include fishing (lobster, bony-fishes and elasmobranch fishing), as well as agriculture. The Wildlife Refuge of Punta Manabique was established as a multi-use protected area with a designated area known as the Maritime Special Use Zone where artisanal fishing is allowed, including the fishing of sharks and rays. Likewise, Livingston serves as the landings center for several smaller fishing communities existing along the coast from the Rio Dulce to the Rio Sarstún that delineates the border with Belize. Key economic activities in Livingston include tourism, fisheries, and agriculture [25].

Local shark fishing is carried out in pelagic waters (90–200 feet). In contrast, ray fishing is carried out in the coastal, shallow waters of the Bahia de Amatique (Fig 1). In El Quetzalito, eight boats target elasmobranchs, fishing seasonally between October and June. In Livingston, the fleet targeting elasmobranchs includes 12 boats, with five boats fishing year-round. All boats are open skiffs with 1–2 outboard engines, mostly 40 horsepower, and are generally staffed by three crew including the captain. During the current study, we identified three different types of elasmobranch fisheries in both communities: 1) a targeted elasmobranch fishery in El Quetzalito, with both longlines (“palangre”), 20–270 type “J” hooks (Size 16/0), and bottom gillnets 500–1000 m wide, consisting of one panel with 3.5 inch mesh size; 2) a multispecies fishery, in Livingston, that includes finfish and elasmobranchs, using a longline with 300–600 hooks (Size 13/0), which is operated according to the seasonal abundance of the species and product demand; and 3) an incidental fishery in Livingston, where elasmobranchs are bycatch of the shrimp fishery.

### Shark landing monitoring

We conducted surveys every two weeks for several months in both fishing communities. In El Quetzalito, we specifically collected data during January–July (years 2015, 2016, and 2017), and October–December (years 2015 and 2016). In Livingston, data were collected between January–June (years 2015, 2016, and 2017). There is a three-month seasonal closure on shark and ray fishing that varies annually. During our study period, the seasonal shark closure was August–September, while the seasonal ray closure was August–October. Additionally, because of limited funding, we were not able to conduct surveys in Livingston from October–December 2016.

We surveyed the same 20 boats in total to determine elasmobranch abundance, catch composition, bait and fishing gear. For each shark landed, the following data were recorded: local name, scientific name, total length (TL), fork length (FL), precaudal length (PCL), and sex. For rays, the following data were recorded: local name, scientific name, sex, disc width (DW) and disc length (DL); TL was also measured for Pseudobatidae. When possible, maturity for males was determined by assessing the calcification and rotation of claspers (e.g. [26,27]). Additional data included type of fishing gear and bait used. All specimens were examined and identified to lowest taxon possible according to Bigelow and Schroeder [19], Compagno [28,29] Garrick [30], Fisher et al. [31] and Compagno et al. [32].

### Data analysis

During the study period we were not able to record measurements of three *G*. *cirratum* individuals. Only measured individuals were recorded in the size composition and sex ratio of landings. Sex-specific size composition was additionally plotted for all species with ≥20 measured individuals, evaluated for normality (Kolmogorov-Smirnov, D) and homoscedasticity (Levene test, F), and compared using Student`s t-test or Wilcoxon rank sum test with continuity correction, as appropriate. All analyses were carried out using the R statistical package [33].

## Results

### Catch composition

During the study period, we recorded landings data for 688 chondrichthyans: 563 sharks, 122 rays and three chimaeras. Captured individuals were represented by two subclasses, eight orders and 13 families (S1 Table). Three families comprised the bulk of the landings. Carcharhinidae accounted for 56% of the recorded catch, while Dasyatidae and Sphyrnidae accounted for 15% and 13%, respectively.

We recorded data for 21 genera and 31 species; eight species are first records in Guatemalan waters (S1 Table). The four most frequent species in the catch composition were the silky shark *Carcharhinus falciformis* (29.9%), the sharpnose sharks *Rhizoprionodon* spp. (12.9%), the scalloped hammerhead shark *S*. *lewini* (12.6%) and the longnose stingray *Hypanus guttatus* (11.9%).

**Fig 2:**
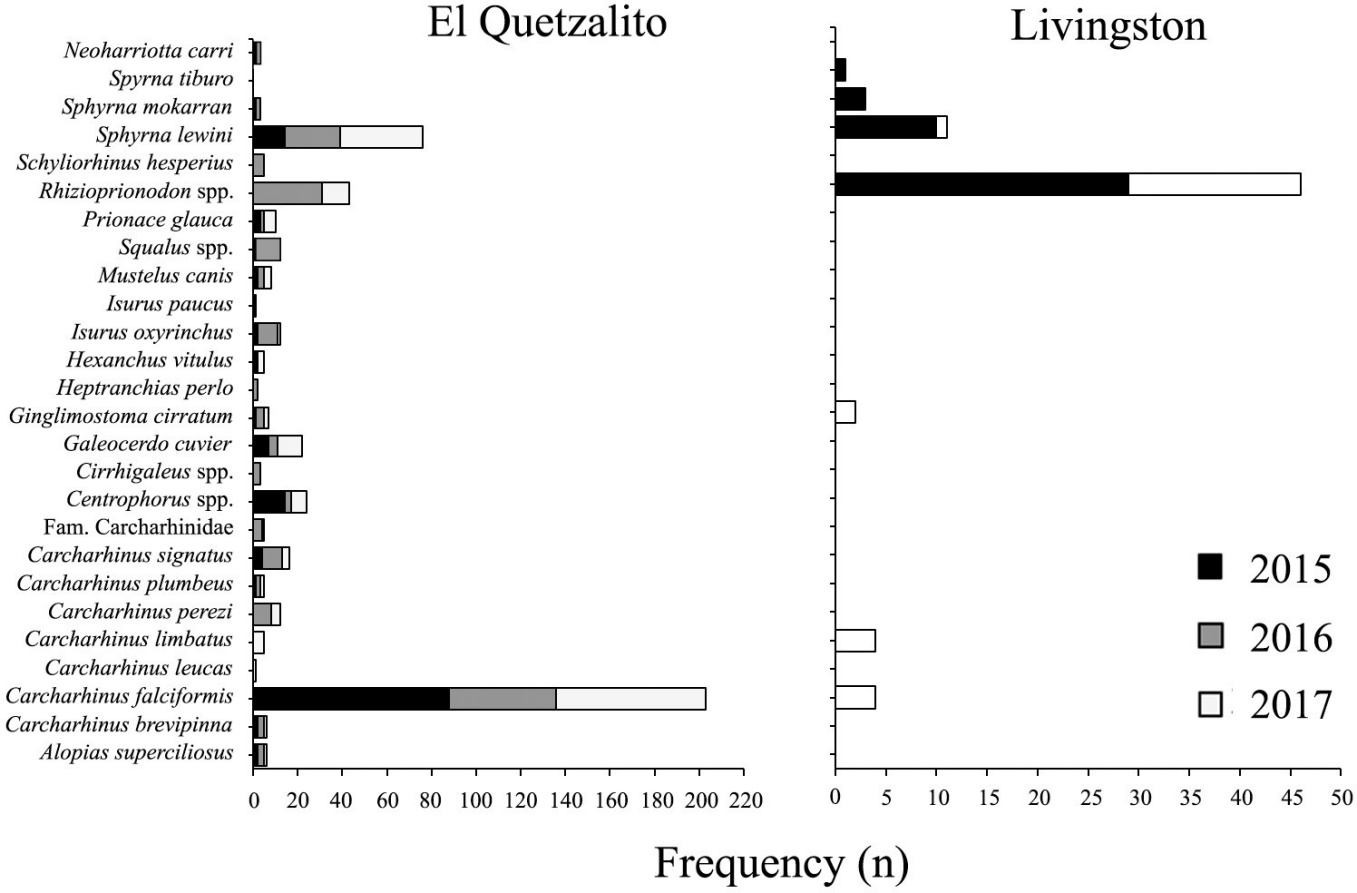
Captures of chimaera and sharks and during the study period, per fishing community and year.

**Fig 3:**
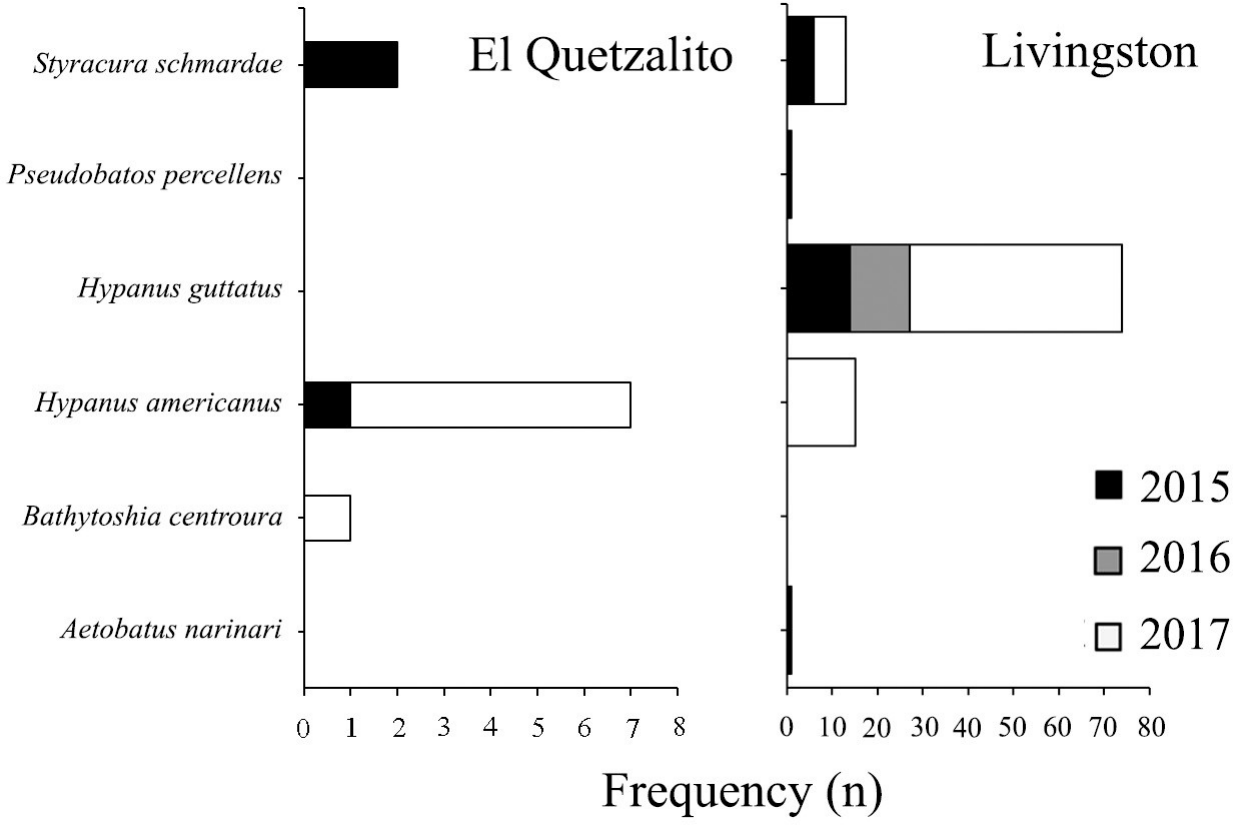
Captures of rays during the study period, per fishing community and year.

Species catch composition varied between both communities, as well as between the years sampled (Figs 2 and 3). In El Quetzalito, we recorded data for 513 chondrichthyans: 24 species of sharks, four rays and one chimaera. The most frequent species in the catch composition were *C*. *falciformis* (39.6%), *S*. *lewini* (14.8%), *Rhizoprionodon* spp., (8.4%), *Centrophorus* spp. (4.7%) and tiger shark *Galeocerdo cuvier* (4.3%) (Table 1). In Livingston, we recorded data for 175 elasmobranchs: eight species of sharks and five species of rays. The most frequently recorded species in the catch composition were *H*. *guttatus* (42.3%), *Rhizoprionodon* spp. (26.3%), southern stingray *Hypanus americanus* (8.6%) and the chupare stingray *Styracura schmardae* (7.4%) (Table 1).

**Table 1:** Catch composition by species, locality, number of individual (n), total length (TL, in cm), disc width (DW, in cm).

Species	n (Overall)	El Quetzalito			Livingston		
		n	Range size (TL/DW)	Mean size ± SD	n	Range size (TL/DW)	Mean size ± SD
**Chimaera**							
*Neoharriotta carri*	3	3	73.0–88.0	78.0 ± 8.7	-	-	-
**Batoids**							
*Aetobatus narinari*	1	-	-	-	1	-	26.0
*Bathytoshia centroura*	1	1	-	158.0	-	-	-
*Hypanus americanus*	22	7	64.0–151.0	90.8 ± 30.2	15	46.0–105.0	74.7 ± 16.0
*Hypanus guttatus*	82	8	55.0–87.0	62.1 ± 10.5	74	36.0–121.0	61.3 ± 16.2
*Pseudobatos percellens*	1	-	-	-	1	-	48.0
*Styracura schmardae*	15	2	100.0–132.0	116.0 ± 22.6	13	74.0–122.0	94.7 ± 15.2
**Sharks**							
*Alopias superciliosus*	6	6	218.0–363.0	303.8 ± 56.4	-	-	-
*Carcharhinus brevipinna*	6	6	111.0–168.0	140.2 ± 25.1	-	-	-
*Carcharhinus falciformis*	207	203	80.0–275.0	138.4 ± 39.3	4	87.0–103.0	97.9 ± 7.3
*Carcharhinus leucas*	1	1	-	215.0	-	-	-
*Carcharhinus limbatus*	9	5	130.0–197.0	168.6 ± 26.4	4	68.0–159.0	93.8 ± 43.6
*Carcharhinus perezi*	12	12	124.0–242.0	182.2 ± 36.0	-	-	-
*Carcharhinus plumbeus*	5	5	177.0–223.0	199.8 ± 21.8	-	-	-
*Carcharhinus signatus*	16	16	100.0–242.0	140.0 ± 38.5	-	-	-
Fam. Carcharhinidae	5	5	146.0–258.0	194.6 ± 49.6	-	-	-
*Galeocerdo cuvier*	22	22	116.0–270.0	204.9 ± 37.7	-	-	-
*Prionace glauca*	10	10	251.0–330.0	287.8 ± 28.1	-	-	-
*Rhizoprionodon* spp.	89	43	55.0–135.0	85.7 ± 15.2	46	45.5–89.0	72.1 ± 12.1
*Isurus oxyrinchus*	12	12	143.0–210.0	179.9 ± 23.1	-	-	-
*Isurus paucus*	1	1	-	260.0	-	-	-
*Sphyrna lewini*	87	76	91.0–282.0	135.6 ± 51.8	11	60.9–162.0	102.5 ± 28.5
*Sphyrna mokarran*	6	3	150.0–242.0	205.3 ± 48.8	3	90.5–140.0	120.8 ± 26.6
*Sphyrna tiburo*	1	-	-	-	1	-	56.0
*Mustelus canis*	8	8	65.0–114.0	85.3 ± 17.0	-	-	-
*Hexanchus vitulus*	5	5	61.0–165.0	135.0 ± 42.0	-	-	-
*Heptranchias perlo*	2	2	28.0–37.0	32.5 ± 6.4	-	-	-
*Centrophorus* spp.	24	24	48.0–159.0	130.9 ± 29.6	-	-	-
*Cirrhigaleus* spp.	3	3	110.0–128.0	117.0 ± 9.6	-	-	-
*Ginglymostoma cirratum*	9	7	171.0–230.0	197.1 ± 21.7	2	-	-
*Scyliorhinus hesperius*	5	5	42.0–51.0	45.0 ± 3.6	-	-	-
*Squalus* spp.	12	12	26.6–54	42.4 ± 8.1	-	-	-

### Size composition–Sharks

**Fig 4:**
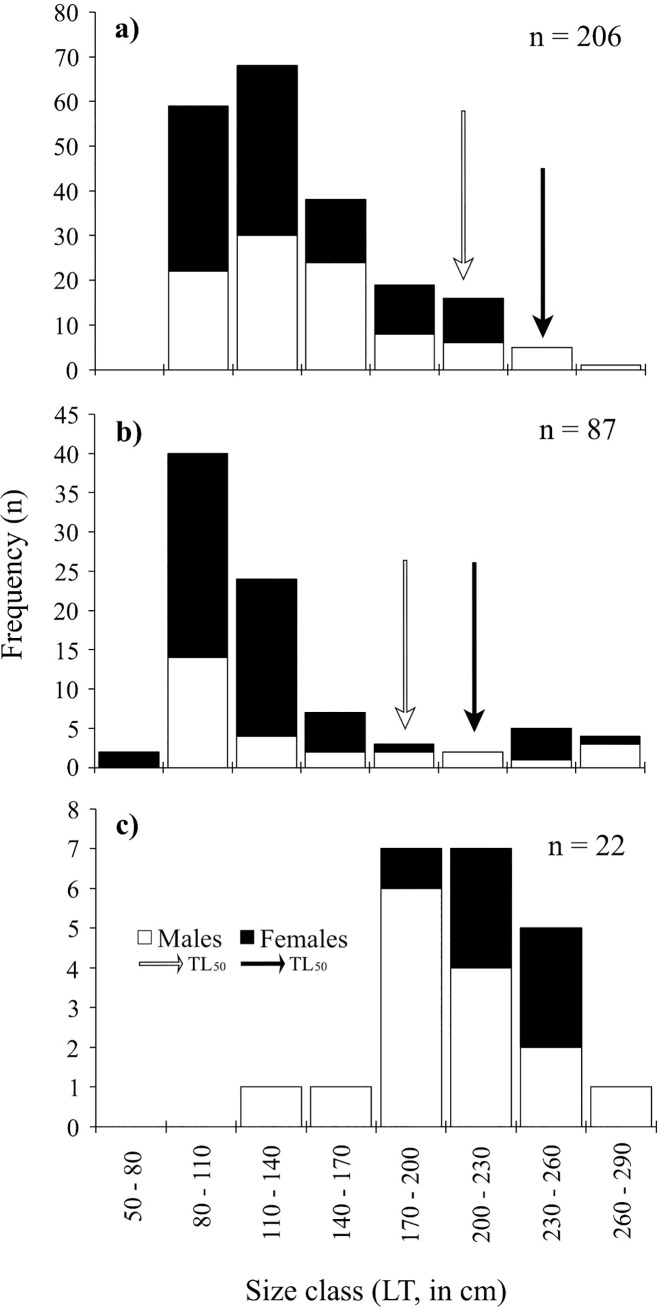
**Size frequency distribution of a) *C*. *falciformis*, b) *S*. *lewini*, c) *G*. *cuvier*, between 2015 and 2017 from landings along Guatemala’s Caribbean coast.** Black arrows represent total length at 50% maturity (TL_50_) for females and white arrow for males, according to Bonfil [34], Branstetter et al. [35] and Bejarano-Álvarez et al. [36].

Sizes of landed sharks ranged from 26.6 cm TL for *Squalus* spp. to 336 cm TL for the bigeye thresher shark *Alopias superciliosus*. The most frequently captured species was *C*. *falciformis*; sizes for this species ranged from 80–275 cm TL. Landings contained more females than males, yielding a ratio of 1.4: 1 (*χ*^*2*^ = 15.78; *P* = 0.00; Table 2). Yet, average size at capture between sexes showed no significant difference (*W* = 4610.5, *P* = 0.092; Table 2; Fig 4A). Among landed specimens in this study (n = 207), 96.6% were juveniles (Fig 4A).

**Table 2:** Chondrichthyes species recorded during the monitoring of landings in two fishing communities along Guatemala’s Caribbean coastline.

Species	Female			Male			Sexual ratio (Female:Male)	Chi (*P-value*)
	n	Size range (TL/DW)	Size mean ± SD	n	Size range (TL/DW)	Size mean ± SD		
**Chimaera**								
*Neoharriotta carri*	2.0	73.0–88.0	80.5 ± 10.6	1.0	-	73.0	2.0:1.0	0.20 (0.65)
**Rays**								
*Aetobatus narinari*		-	-	1.0	-	26.0	-	-
*Bathytoshia centroura*	1.0	-	158.0		-	-	-	-
*Hypanus americanus*	16.0	61.0–108.0	80.3 ± 13.7	6.0	46.0–151.0	78.5 ± 38.6	2.7:1.0	4.55 (0.03)
*Hypanus guttatus*	47.0	36.0–106.5	65.0 ± 15.2	35.0	44.5–121.0	56.5 ± 15.3	1.3:1.0	1.76 (0.19)
*Pseudobatos percellens*		-	-	1.0	-	48.0	-	-
*Styracura schmardae*	9.0	74.0–132.0	96.9 ± 17.8	6.0	75.0–122.0	98.5 ± 17.3	1.5:1.0	0.60 (0.44)
**Sharks**								
*Alopias superciliosus*	3.0	305.0–363.0	338.3 ± 29.9	3.0	218.0–334.0	269.3 ± 59.1	1.0:1.0	0.00 (1.00)
*Carcharhinus brevipinna*	2.0	111.0–133.0	122.0 ± 15.6	4.0	112.0–168.0	149.3 ± 25.3	0.5:1.0	0.67 (0.41)
*Carcharhinus falciformis*	110.0	80.0–229.0	133.1 ± 36.0	97.0	86.0–275.0	142.8 ± 42.3	1.1:1.0	0.82 (0.37)
*Carcharhinus leucas*		-	-	1.0	-	215.0	-	-
*Carcharhinus limbatus*	6.0	68.0–184.0	122.0 ± 55.6	3.0	130.0–197.0	162.0 ± 33.6	2.0:1.0	1.00 (0.32)
*Carcharhinus perezi*	2.0	124.0–155.0	139.5 ± 21.9	10.0	135.0–242.0	190.7 ± 32.4	0.2:1.0	5.33 (0.02)
*Carcharhinus plumbeus*	1.0	-	223.0	4.0	177.0–220.0	194.0 ± 20.2	0.3:1.0	1.8 (0.18)
*Carcharhinus signatus*	9.0	100.0–242.0	138.0 ± 46.8	7.0	117.0–198.0	142.6 ± 28.0	1.3:1.0	0.25 (0.62)
*Carcharhinus* spp.	3.0	146.0–234.0	187.3 ± 44.2	2.0	153.0–258.0	205.5 ± 74.2	1.5:1.0	0.20 (0.65)
*Galeocerdo cuvier*	7.0	180.0–255.0	225.0 ± 23.3	15.0	116.0–270.0	195.6 ± 40.1	0.5:1.0	2.91 (0.09)
*Prionace glauca*		-	-	10.0	251.0–330.0	287.8 ± 28.1	-	-
*Rhizoprionodon* spp.	69.0	45.5–135.0	81.0 ± 15.5	20.0	48.5–94.0	71.3 ± 10.9	3.2:1.0	26.98 (0.00)
*Isurus oxyrinchus*	5.0	160.0–207.0	179.4 ± 22.2	7.0	143.0–210.0	180.3 ± 25.5	0.7:1.0	0.33 (0.56)
*Isurus paucus*	1.0	-	260.0		-	-	-	-
*Sphyrna lewini*	59.0	60.9–260.0	124.7 ± 43.8	28.0	91.0–282.0	145.5 ± 61.0	2.1:1.0	11.05 (<0.01)
*Sphyrna mokarran*	4.0	90.5–224.0	146.6 ± 55.9	2.0	150.0–242.0	196.0 ± 65.1	2.0:1.0	0.67 (0.41)
*Sphyrna tiburo*		-	-	1.0	-	56.0	-	-
*Mustelus canis*	4.0	68.0–114.0	88.0 ± 21.1	4.0	65.0–95.0	82.5 ± 57.8	1.0:1.0	0.00 (1.00)
*Hexanchus vitulus*	2.0	61.0–165.0	113.0 ± 73.5	3.0	144.0–153.0	149.7 ± 4.9	0.7:1.0	0.2 (0.65)
*Heptranchias perlo*	2.0	28.0–37.0	32.5 ± 6.4		-		-	-
*Centrophorus* spp.	23.0	48.0–159.0	133.0 ± 28.3	1.0	-	82.0	23.0:1.0	20.17 (<0.01)
*Cirrhigaleus* spp.	3.0	110.0–128.0	117.0 ± 9.6		-	-	-	-
Ginglymostoma cirratum*	3.0	171.0–200.0	183.0 ± 12.9	3.0	200.0–230.0	216.0 ± 15.1	1.0:1.0	0.00 (1.00)
*Scyliorhinus hesperius*	5.0	42.0–51.0	45.0 ± 3.6		-	-	-	-
*Squalus* spp.	4.0	26.6–54.0	40.4 ± 14.6	8.0	39.0–49.0	43.4 ± 3.0	0.5:1.0	1.33 0.25)

Species listed include data on total number, sex, size (range, average and standard deviation), sexual ratio and Chi-square.

*Three specimens were identified but could not be sexed

The maximum size of *S*. *lewini* recorded in this study was 282 cm TL; size range was 60.9–282 cm TL. Females were more frequent in landings, with no significant difference between catch size (*W* = 732, *P* = 0.395; Table 1, Fig 4B) between males and females. Among landed specimens in this study (n = 87), 85.1% were juveniles (Fig 4B).

The maximum size of *G*. *cuvier* was 270 cm TL; size range was 116–270 cm TL. Size composition between females and males revealed that males were more frequently captured (Table 1). Size composition between sexes was statistically different (*W* = 79, *P* = 0.066), where females were larger (Table 2; Fig 4C). Among landed specimens in this study (n = 22), all individuals (15 males and seven females) were juveniles (Fig 4C).

**Fig 5:**
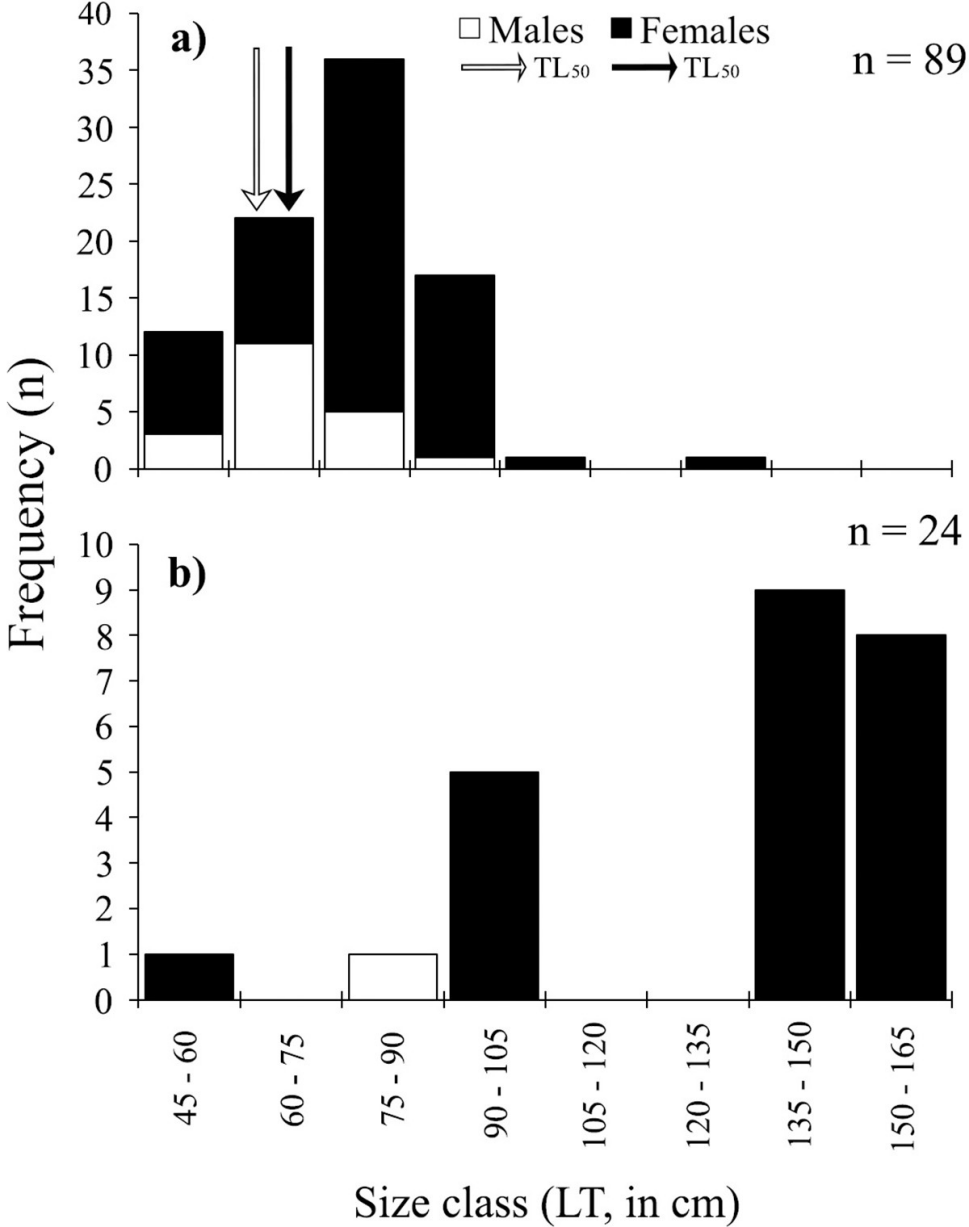
**Size frequency distribution of a) *Rhizoprionodon* spp., b) *Centrophorus* spp., between 2015 and 2017 from landings along Guatemala’s Caribbean coast.** Black arrows represent total length at 50% maturity (TL_50_) for females and white arrow for males, according to Carlson and Baremore [37], Mattos et al. [38] and Motta et al. [39].

The maximum size of *Rhizoprionodon* spp. recorded in this study was 135 cm TL with captures ranging in size from 45.5–135 cm TL. Landings contained fewer males of smaller size than females (Table 1) (*W* = 161, *P* = 0.04; Table 1; Fig 5A). Among landed specimens in this study (n = 89), only 13.5% were juveniles (Fig 5A).

Finally, the maximum size of *Centrophorus* spp. recorded in this study was 159 cm TL, with sizes ranging from 48–159 cm TL. Among landed specimens in this study (n = 24), 23 individuals were females; only one male specimen was recorded (Table 1, Fig 4B). Landing and composition showed that captures were dominated by females (Table 1; Fig 5B). Additionally, four females were pregnant (size range 146–155 cm TL).

### Size composition–rays

**Fig 6:**
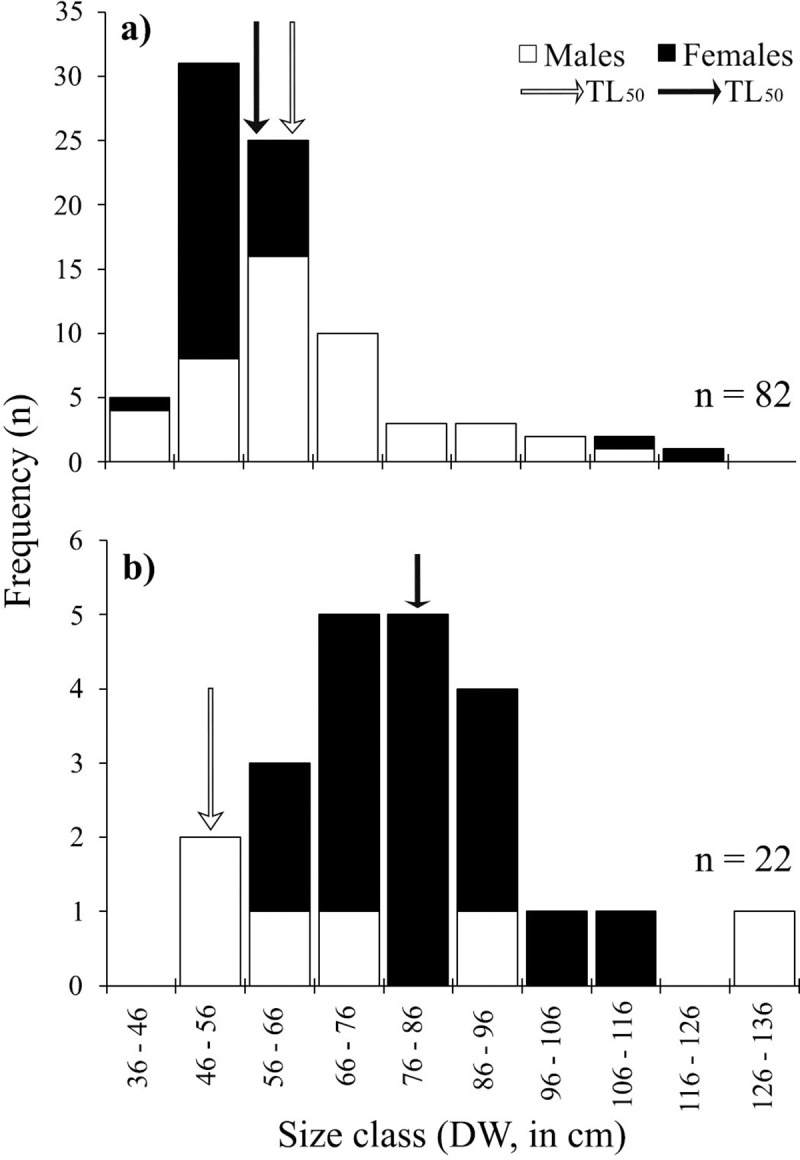
**Size frequency of a) *H*. *guttatus* and b) *H*. *americanus* recorded between 2015 and 2017 from landings along Guatemala’s Caribbean coast**. Black arrows represent total length at 50% maturity (TL_50_) for females and white arrow for males, according to Ramírez-Mosqueda et al. [40] and Tagliafico et al. [41].

The maximum size of *H*. *guttatus* recorded in this study was 121 cm DW, size range was 36–121 cm DW. Number and size composition by sex showed females dominated the captures (Table 1). Average size of capture between females and males was significantly different (*W* = 1198, *P* = <0.01; Table 1; Fig 6A). Among landed specimens in this study (n = 82), 59.1% were juveniles.

The maximum size of *H*. *americanus* recorded in this study was 151 cm DW, size range was 46–151 cm DW. Landings and size composition by sex showed females dominated the captures (Fig 5B). Yet, average size capture between females and males showed no significant difference (*W* = 61.5, *P* = 0.338; Table 1; Fig 6B). Among landed specimens in this study (n = 22), 40.9% were juveniles.

### Chondrichthyans capture rate, according to fishing gear

**Fig 7:**
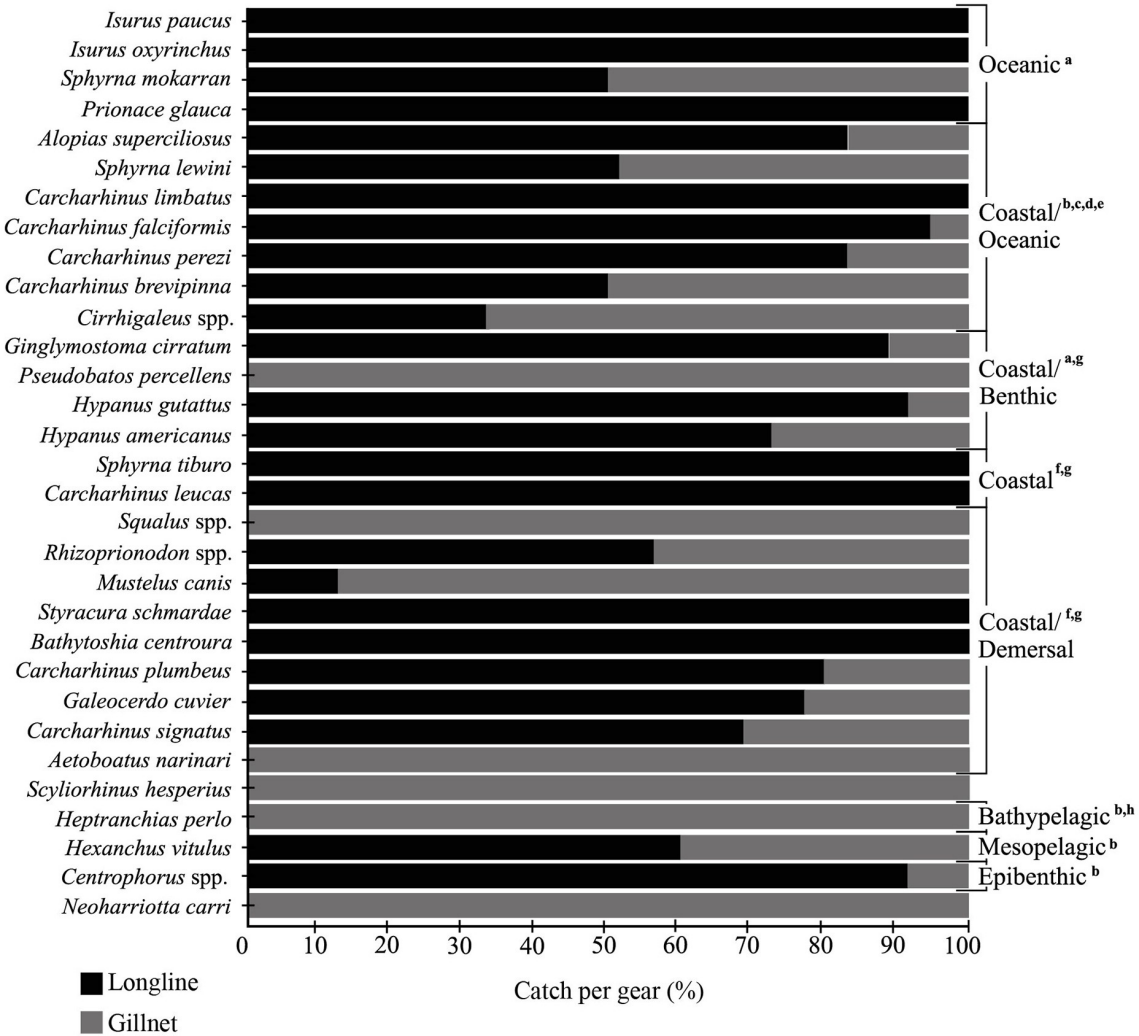
Fishing gears used to catch Chondrichthyan fish landed in this study. Data were grouped according to the following authors: a. Compagno [28], b. Compagno [29], c. Estupiñan-Montaño et al. [42], d. Estupiñan-Montaño et al. [43], e. Estupiñan-Montaño et al. [44], f. Estupiñan-Montaño et al. [45], g. Carpenter [46] and Ebert [47].

During the study period, longlines were used to land 76.3% of all chondrichthyans (n = 525), followed by gillnets (23.7%, n = 163). Shark captures were dominated by longline use for 74.2% of landings (n = 418), followed by gillnets (25.8%, n = 45). Specifically, for the three most captured sharks, 94.7% individuals of *C*. *falciformis* were captured with longlines and 5.3% with gillnets. For *S*. *lewini*, 51.7% individuals were captured with longlines and 48.3% with gillnets. For *Rhizoprionodon* spp., 43.8% individuals were captured with gillnet and 56.2% with longline (Fig 7). Similarly, for the capture of rays, longlines were more frequently used (87.7.6%, n = 107), followed by gillnets (12.3%, n = 15). As for the most captured rays, specifically for *H*. *guttatus*, 91.4% individuals were captured with longlines and 8.6% with gillnet. For *H*. *americanus*, 72.7% individuals were captured with longlines and 27.3% with gillnet. For the capture of the chimaeras, gillnet was the only fishing gear used (n = 3) (Fig 5). Finally, results show there was no correlation between habitat and fishing gear (Fig 7).

## Discussion

This study reports the first fisheries landings analysis of sharks, rays and chimaeras along Guatemala’s data-poor Caribbean coast. Over the course of a three-year study (2015 to 2017), we recorded data on 688 specimens, represented by 31 chondrichthyan species. The broad diversity of species reflects the variety of habitats present in the fishing zones including coastal, coral reef, pelagic and deep-sea habitats. At least eight species recorded during this study are new records for Guatemala´s Caribbean Sea (S1 Table), with 22 previously recorded in the MAR region (e.g. [9,48,49]). Additionally, landings monitoring in this area has resulted in at least eight new species records for several deep-sea chondrichthyes species in Guatemala´s Caribbean Sea such as: *Hexanchus vitulus*, *Centrophorus* spp., *Cirrihigaleus* spp., *Squalus* spp., etc. (this study; [20–22]). Although deep-sea sharks are not regularly targeted by fishers, they are occasionally caught. If captured, fishers may utilize the meat, or render the liver for shark oil, depending on the species’ size (e.g. *Squalus* spp., *Scyliorhinus hesperius*, *Neharriotta carri*, *Heptranchias perlo* are discarded due to their small size capture). Additional studies are needed to identify the behavior and ecology of deep-sea species in the Caribbean, especially for data-poor species, in light of increasing fisheries effort.

The most frequently captured species recorded in the current study, *C*. *falciformis* and *S*. *lewini*, possess size ranges similar to those described for the species in the MAR region [15,36,48]. In contrast, the size range recorded for landed *G*. *cuvier* were smaller (male: 270cm TL, female: 255 cm TL) than that recorded by Branstetter et al. [35] in the Gulf of Mexico (male: 340 cm TL, female: 381 cm TL). Additionally, we recorded three female *Cirrhigaleus* spp. (size range was 110–128 cm TL). Morphometric analysis conducted on these specimens suggest they could belong to *Cirrhigaleus asper*. Further DNA analysis will determine if these specimens belong to *C*. *asper* or represent new species. Morphometric analysis on one *Cirrhigaleus* spp. specimen (female, 128 cm TL) exceeds the maximum size recorded for *C*. *asper* (123.5 cm TL; [50]), making this specimen the largest recorded to date, based on morphometric data collected.

In this study, size frequency analysis of captured species highlights landings dominated by juvenile sharks, data that complement several other studies on artisanal and industrial shark fisheries that record a high catch of juveniles (e.g. [3,51]). Powers et al. [52] examined records from organized recreational shark fishing (fishing rodeos) in the northern Gulf of Mexico to establish that the size of large sharks has decreased by 50–70% since 1980. The reduction in the occurrence and sizes was greatest for *G*. *cuvier* and *C*. *leucas*, and to a lesser extent for hammerhead sharks *Sphyrna* spp. Although our study’s data set does not allow determination of fishing-induced reduction in shark size, we have established reference baselines for future regional studies. During the study period, according to data collected on reproductive condition of female sharks landed, most individuals of *C*. *falciformis*, *S*. *lewini*, *G*. *cuvier*, *C*. *perezi*, *C*. *signatus*, were sexually immature. The high prevalence of juveniles and immature individuals within recorded captures suggests the fisheries areas may serve as a nursery area. Additional research on the habitat use and reproductive status of these species in the Caribbean region of Guatemala will be needed to confirm the existence of a critical nursery habitat. Studies on habitat use of sharks and rays along the MAR have revealed sharks may utilize oceanic atolls and inshore locations as breading and nursery areas [10,53]. In Belize, Pikitch et al. [10] suggest that Glover’s Reef atoll is used for purposes of breeding based on the presence of neonate and small juvenile *G*. *cirratum*, *C*. *perezi*, *Negaprion brevirostris*, and *H*. *americanus*. These authors suggest that some of the other unexplored oceanic atolls and inshore areas along the MAR and Belizean coast may also support breeding areas for other sharks and rays.

Sexual segregation in habitat preferences, and reproductive or foraging behavior are considered characteristic of elasmobranch populations [54], and have been recorded for several shark species [55,56]. This study also revealed patterns of sexual segregation across several shark species. Sex ratios were female skewed for the captures of *Centrophorus* spp., *S*. *lewini*, *S*. *mokarran*, *Rhizoprionodon* spp., *H*. *americanus*, while a higher ratio of males was recorded for *P*. *glauca*, *Squalus* spp., *S*. *hesperius*. Differences in sex ratios have also been attributed to seasonality, fishing gear, and fishing location [57]. Species-specific informations about populations and notably sex-mediated movement patterns of fished chondrichthyans in the Western Caribbean is limited outside of studies conducted on whale sharks [13,58], manta rays [59], Caribbean reef sharks [10,60], and great hammerheads [61]. This study highlights the need to conduct more regionally-specific research to improve our understanding of sex-mediated shark spatial ecology.

This study’s landings monitoring highlighted three types of fisheries that take place in the study area: 1) a targeted shark fishery, 2) multi-taxa, and 3) incidental capture. The targeted shark fishery takes place only in El Quetzalito. In this location, the main fishing gear used for the capture of sharks are longlines, followed by gillnets. Results from this study show large sharks (e.g. *Carcharhinus* spp., *Sphyrna* spp., *P*. *glauca*, *G*. *cuvier*, etc.) are captured using these two fishing gears (Fig 4). In Livingston, the fishery is multi-taxa and fishing gear used is mainly longline (locally known as “cimbra”). Main captures include small sharks (e.g. *Rhizoprionodon* spp.) and rays (e.g. *H*. *guttatus* and *H*. *americanus*) (Fig 4), but may also include other species, such as catfish *Bagre marinus* and channel catfish *Ictalurus punctatus*, occasionally tarpon *Megalops atlanticus* and species of the snapper family Lutjanidae (snappers). In El Quetzalito, tarpon is mainly captured for bait when fishing for sharks, whereas in Livingston, tarpon is captured for local consumption, while elasmobranchs, notably ray species form the bycatch of an extensive shrimp fishery [62]. Considerable debate surrounds the sustainability of shark fisheries globally, with suggested measures to attain sustainability (e.g. the identification of marine protected areas that protect critical shark habitat and populations, regulations that control the number of hooks, gear modification to reduce bycatch, switching fishing to areas where lower bycatch per unit effort has been recorded, closed seasons and no-take areas, etc.) [63–65]. However, there are a few examples in Latin American or African shark fisheries of these measures being implement due to a paucity of data on which to base them.

Restoring populations of sharks where juveniles are mostly captured will prove challenging in Guatemala as long as fisheries in the study area are based on a combination of longlines and gillnets that effectively capture all sizes and species of sharks and rays, notably in coastal waters predominantly inhabited by juveniles of coastal elasmobranch species. Also, due to a lack of historical landings data, it is not possible to determine if the greater proportion of juveniles captured is due to overfishing of larger individuals or changes in gear, although Belizean and Guatemalan fishers note anecdotally a broad decline of large sharks and ecological extinction of formerly abundant species since the 1980s [16,60]. Continued monitoring of these areas, and additional studies encompassing age and growth, reproduction and feeding ecology of sharks and rays, along with socio-economic studies on catch and effort trends and product value and trade flow, will help to further characterize the populations and enable authorities to establish appropriate management and protection measures for threatened species and define the feasibility of a sustainable shark fishery.

The majority of immature *C*. *falciformis* and *S*. *lewini* landed is cause for concern, undermining the persistence and sustainability of the fisheries. Moreover, *S*. *lewini* is listed as Endangered by the International Union for Conservation of Nature [66,67] and *C*. *falciformis* is listed as Vulnerable due to continued declines in populations globally [68]. Both species are further listed under Appendix II of CITES and Appendix I and II of the Convention on Migratory Species. Guatemala is a contracting party of CITES and must therefore meet admissions requirements to certify the export of products and by-products of elasmobranchs. CITES specifically requires the development of a Non-Detrimental Findings (NDF) to assure that trade is not adversely impacting populations, which has not yet been conducted for elasmobranch species in Guatemala. To meet NDF and CITES requirements, first a document verifying fisheries landings would need to be issued by the Managing Authority of Fisheries (Directorate of Fisheries and Aquaculture Regulations—DIPESCA). This process is challenging in El Quetzalito due to its isolation. In Livingston, a lack of trained and authorized staff limits landings verification. Government entities regulating fisheries don't have the personnel required to verify landings, with only two staff available to monitor Guatemala’s Caribbean region. Considering that half of the shark catch consists of CITES-listed species, it would behoove the Government to develop a monitoring program that guarantees traceability and control of onward trade for implementing CITES regulations to ultimately define whether the fishery is sustainability or requires further management and conservation measures.

Several conservation measures have been enacted in Guatemala that including a one to three-month seasonal closure on shark and ray fishing that varies annually and is set by fishers (e.g. Acuerdo Ministerial 42–2011; Acuerdo Ministerial 43–2012; Acuerdo Ministerial 33–2013). Initially, the seasonal fishing closure was set during the months of August-September, when alternative fisheries are available (e.g. lobster) and during the peak of the hurricane season. However, conservation measures enacted in Guatemala need to be revised, as they were proposed without scientific information (as there was none available at that time). There is no historical information regarding the elasmobranch fishery in the area. Therefore, putting these measures within the context of our results is not possible. Our study provides valuable information, which can be used to revise or propose different measures for the use and management of the shark and ray fishery in the area. In 2011, the Central American Fisheries and Aquaculture Organization passed a regional ban (OSP-05-11) on shark finning, requiring all fishers to land sharks whole. These measures have been complemented by the FAO catalyzed national plan of action for sharks initiative (NPOA). Guatemala’s Fisheries and Agriculture Ministries drafted the country’s NPOA in 2008 [69]. However, the NPOA remains data-less, without identified priorities, research gaps or policies that would ensure that the plan’s objectives are carried out. Moreover, the lack of governance, protection and patrolling to regulate fishing activities in the area, reduce the transboundary fishing in Belize and Honduras’ territorial waters [70], combined with a continued lack of funding for enforcement agencies are limiting factors that strongly undercut the effective management of shark and ray fisheries and the conservation of threatened chondrichthyans in Guatemala and neighboring territorial seas.

Results presented in this study represent a baseline of information on shark and ray diversity, highlighting the capture of low productivity juveniles of threatened elasmobranch species, and an increase in the number of known shark, ray and chimaera species in Guatemala from 22 to 31. Study results further expand current knowledge of elasmobranch exploitation and traditional fisheries in Guatemala´s Caribbean Sea, and highlight the key obstacles to sustainable shark fisheries. Continued community-based landings monitoring of elasmobranch fisheries are needed to characterize changes in fishing effort and shifts in species captured while community-based projects are developed to redirect and reduce fishing effort and stem declines in Guatemala’s fish populations.

## Supporting information

S1 Table Chondrichthyes taxa recorded in landings monitoring, conducted in two fishing communities of the Caribbean of Guatemala. Taxonomic classification according to Ebert et al. [71], Carvalho et al. [72], Last et al. [73, 74]. *indicates species recorded for the first time in the Caribbean of Guatemala.
(DOCX)
