# Supplementary material for: Description and characterization of the artisanal elasmobranch fishery on Guatemala’s Caribbean coast
Source: PLoS One. 2020 Jan 13;15(1):e0227797. doi: 10.1371/journal.pone.0227797 (PMC6957299; doi:10.1371/journal.pone.0227797)
Supplement: S1 Table — Taxonomic classification according to Ebert et al. [71], Carvalho et al. [72], Last et al. [73, 74]. *indicates species recorded for the first time in the Caribbean of Guatemala. (DOCX) [file pone.0227797.s001.docx]

**S1 Table. Chondrichthyes taxa recorded in landings monitoring, conducted in two fishing communities of the Caribbean of Guatemala.** Taxonomic classification according to Ebert et al. [34], Carvalho et al. [35], Last et al. [73, 74]. *indicates species recorded for the first time in the Caribbean of Guatemala.

| Subclass | Suborder | Order | Family | Genus | Species | common name | local name |
| --- | --- | --- | --- | --- | --- | --- | --- |
| Holocephali | - | Chimaeriformes | Rhinochimaeridae | *Neoharriotta* | *N. carri* | dwarf sicklefin chimaera | Tiburón elefante |
| Elasmobranchii | Batoidea | Myliobatiformes | Myliobatidae | *Aetobatus* | *A. narinari* | Spotted eagle ray | Raya gavilán |
|  |  |  | Dasyatidae | *Hypanus* | *H. americanus* | Southern stingray | Raya látigo |
|  |  |  |  |  | *H. guttatus* | Longnose stingray | Raya narizona |
|  |  |  |  | *Bathytoshia* | *B. centroura** | Roughtail stingray | Raya narizona |
|  |  |  |  | *Styracura* | *S. schmardae* | Chupare stingray | Raya cachetona, sapa |
|  |  | Rajiformes | Rhinobatidae | *Pseudobatos* | *P. percellens** | Southern guitarfish | Raya guitarra |
|  | Selachii | Carcharhiniformes | Carcharhinidae | *Carcharhinus* | *C. brevipinna* | Spinner shark | Tiburón arenero |
|  |  |  |  |  | *C. limbatus* | Blacktip shark | Tiburón puntas negras |
|  |  |  |  |  | *C. falciformis* | Silky shark | Tiburón catrin, sedoso |
|  |  |  |  |  | *C. perezi* | Reef shark | Tiburón avionsito |
|  |  |  |  |  | *C. plumbeus* | Sandbar shark | Tiburón boleado |
|  |  |  |  |  | *C. signatus** | Night shark | Tiburón payaso |
|  |  |  |  |  | *C. leucas* | Bull shark | Tiburón toro |
|  |  |  |  | *Galeocerdo* | *G. cuvier* | Tiger shark | Tiburón tigre |
|  |  |  |  | *Prionace* | *P. glauca* | Blue shark | Tiburón azul |
|  |  |  |  | *Rhizoprionodon* | *Rhizoprionodon* spp*.* | sharpnose shark | Cazón |
|  |  |  | Scyliorhinidae | *Scyliorhinus* | *S. hesperius* | Whitesaddled catshark | Tiburón leopardo |
|  |  |  | Sphyrnidae | *Sphyrna* | *S. lewini* | Scallop hammerhead shark | Tiburón martillo, tintorera |
|  |  |  |  |  | *S. mokarran* | Great hammerhead shark | Tiburón martillo, tintorera |
|  |  |  |  |  | *S. tiburo* | Bonnethead shark | Tiburón de pala |
|  |  |  | Triakidae | *Mustelus* | *M. canis* | Dusky smooth-hound Shark | Tiburón larguirin |
|  |  | Hexanchiformes | Hexanchidae | *Heptranchias* | *H. perlo* | Sharpnose seven gill shark | Tiburón cañabota, |
|  |  |  |  | *Hexanchus* | *H. vitulus** | Atlantic sixgill shark | Tiburón cañabota, limón |
|  |  | Lamniformes | Alopiidae | *Alopias* | *A. superciliosus* | Bigeye thresher shark | Tiburón zorro |
|  |  |  | Lamnidae | *Isurus* | *I. paucus** | Mako shark | Tiburón perra |
|  |  |  |  |  | *I. oxyrinchus* | Longfin mako shark | Tiburón perra oscura |
|  |  | Squaliformes | Centrophoridae | *Centrophorus* | *Centrophorus* spp*.** | Gulper shark | Tiburón espinoso |
|  |  |  | Squalidae | *Cirrhigaleus* | *Cirrhigaleus* spp*.** | Spurdog | Tiburón espinudo |
|  |  |  |  | *Squalus* | *Squalus* spp.*** | dogfish | Tiburón espinudo |
|  |  | Orectolobiformes | Ginglymostomatidae | *Ginglymostoma* | *G. cirratum* | Nurse shark | Tiburón gata, nodriza |
